# Disquisition on the Application of the Principles of Cure in Those Fevers, Attributed to Marsh Miasmata, on the Hypothesis of the Abstraction of Oxygen-Gas from the Atmosphere, as the Cause of Them

**Published:** 1807-05

**Authors:** 


*Disquisition on the Application of the Principles of Cure in those Fevers, attributed to Marsh Miasmata, on the Hypothesis of the Abstraction of Oxygen-Gas from the Atmosphere, as the Cause of them.*

TO doctrines of an innovating nature are generally opposed, inveterate prejudices, preconceived hypotheses, dogmatical assertions, or sophisticated reasonings: the sullen pride of man hath from the above arsenal, clad him with a coat of mail more invulnerable than that fabled of Pelides.—When, however, it shall be recollected, that at a period sometime antecedent to this, the generation of fevers by Marsh Miasmata was treated with a succinctness of argument, an accuracy of remark, and a diligence of experiment, highly laudable; surely, nothing of an innovating spirit can be found in this attempt. My object, after cursorily stating and reviewing this theory, is to establish it on a firmer basis, and to demonstrate its perfect coincidence with the principles of the animal œconomy.

Our first step is the analysis of the atmosphere where marsh miasmata prevail. To medical and philosophical readers, it will be needless to notice the division of air by Lavoisier; I shall therefore confine myself to the experiments of Mr. Vanbreda, as given in Mr. Wm. Murray's paper in the Philosophical Transactions of the American Society; and which bear a more immediate reference to the points here to be urged. Mr. Vanbreda discovered by the test of the eudiometer, that the atmosphere of marshes, during the autumnal season, consisted of fourteen or fifteen parts of oxygen, eighty-four or five parts of azote; and that the bulk was made equal by a certain quantity of carbonic gas, a small portion of hydrogen and ammoniacal gases, or aëriform fluids. That these have no share in generating intermittents is inferable from the underwritten considerations. The carbonic gas, when concentrated,

 destroy destroys life; the hydrogen gas begets no disease; the ammoniacal gas, owing to the union of the hydrogen evolved in the putrefactive fermentation with the superabundant azote of the atmosphere, is known to have a most stimulating effect; the azote, in its proportion to the foregoing airs, has no share in destroying life, for the heart immersed in its gas, will retain its irritability several hours. We must here then stop, and look round for other causes to which we attribute the effects of insalubrious situations; the abstraction of oxygen gas, strikes irresistably upon our minds.

The leading feature in this Essay, is to show the curative mode of intermittents on the hypothesis of the abstraction of oxygen being the cause of them. And here it behoves me to apply it to the best grounded theory of fevers of the intermitting class, or, in other words, to the laws of the animal system, as illustrated by the arguments of Darwin. I must suppose every medical reader acquainted with the language of the Zoonomia; since, without such an anticipation, the very elucidating of the explicative terms, would swell this paper to a volume. When the torpor of any part of the system, owing to deficient irritation, occasioned either by the subduction of the natural stimuli, and consequent accumulation of sensorial power; or by the application of powerful stimuli, and consequent exhaustion of the same living principle, what follows? The next link of the tribe of associate motions, falls also into a torpor from defect of the excitement of the sensorial power of association; and so the subsequent one, till a general torpor affects the whole system. This constitutes the cold paroxysms of fever.—This general torpor remains, till the accumulation of the sensorial power has been formed, which may overbalance the defect of excitement of association, and then the torpor ceases, and the hot fit of fever is produced.

The extreme vessels, on the attack of an intermittent, are powerless and atonic; and is not here an evident torpor occasioned by the subduction of the natural stimuli, and consequent accumulation of sensorial power? for were there any subtraction of the latter, the parts would remain paralysed during the action of the vis medicatrix naturæ, and I need scarcely, for better evidence, notice the strong pulse during this very agency. The blood would be absolutely incapable of its circulatory process through the heart, were it not for the admission of oxygen. With the justice of truth, and the justness of reasoning, I think I may may assert, having in full comprehension the foregoing observations, that the torpor, from defect of excitement of the sensorial power of association, occasioning inactivity of the vessels, proceeds from the absence of the same principle of vitality. The stimulus of the blood is decreased, its future impetus increased, by the absence of oxygen. Upon the subduction of this aëriform matter, the sensorial power of irritation is formed, and the circles of irritative associate motions becoming successively affected, an uniform accumulation accrues; hence this principle being restored, or the vis medicatrix naturæ, acting by a distended force in lieu of it, overcomes the defective excitement, and restores the sensorial energy.

Allow me further, (however irrelevantly) to impress my ideas on this head, by adverting to the purification of ships. The smoke arising from the ignited layers of sulphur and charcoal, or from tobacco alone, is too constantly employed for this purpose. A ship of war, on the coast of Guinea, lost sixty of her men from the injuries of a tainted atmosphere, while a sloop, in company with her, had not a single valetudinarian, from the circumstance of the fire-place being on a level with the deck. Now, to account for this salutariness, we must necessarily suppose purifications by the fire, which were preservatives against the baneful attacks of the contaminated atmosphere; these properties must have had their essence in oxygen.

Dr. Fordyce has given his opinion, that the impure air owes its renovation to the current of it produced by burning bodies; to this, the dreadful Harmattan yields its malignity. The Samiel wind, constituted by the pestiferous vapour arising in hot weather from a sandy soil, proves, over the extensive deserts of Asia and Africa, instantly fatal to man and beast; but when it passes over a soil covered with grass and fresh vegetables, its effects are instantly mitigated. How are they mitigated? Let the grass and the vegetables proclaim.—When between the tropic of Cancer and the tropic of Capricorn, the European feels the evening damp, the unlettered Indian bears him to his hut, places him in a smoke, and cures him. Here, speaking in the language of Darwin, the circles of irritative associate motions are overcome, and the awakened association of sensorial power restores the equilibrium.

Sydenham has told us, that the principal curative indication in the plague, was to procure an universal sweat; and Dr. Cullen, as a theorist, on this ground, has cautioned us to allow of the violence of the re-action. Dr. Russel, sel, however, who speaks from actual observation and a lucid mind, holds an opinion diametrically opposite to Dr. Cullen, as is inferable by his admission of venæsection. That this operation, in this disease, upon the strict letter of medical erudition, may be at some times admissible, there can be but slight doubt; but the formidable lancet, used indiscriminately, would mark the foot-steps of its bold employer in death and desolation. In its nature the plague has been defined a pestilential contagion, and bleeding, where the system teems with putrescent matter, seems to require all the boundaries of prudence, all the aids of unerring discretion. Of late it has been written, that this pestiferous disease is not communicable by the malignity of the atmosphere, but by actual contact alone. That this first cause is highly productive of this terrific engine of human destruction, of this assailant of the fair catenation of society, no man can doubt.

Returning from this digressive but collaterally connected matter, I shall proceed to remark on the conformity of medical practice to the ideas I have here been attempting to establish. The practitioner (as if by mechanism) thinks of nothing but giving an emetic before the paroxysmal accession, and copious draughts of bark in the intervals. —If any man ask himself the modus operandi of these medicines, he will immediately give him for answer, the first acts by relaxing the atony and spasm on the extreme vessels; the second, by conferring additional vigour on the system to re-act; and by assisting the heart's efforts arising from the stimulus of distension, owing to accumulated blood, to shake off the incarcerating load on the surface.

That there exists a multitude of diseases, whose causes extend both over the sober and speculative bounds of human cognizance, I do not for a moment deny; but, that the origin of an intermittent is evident by the proper exercise of that cognizance, I am certainly disposed to think, and I hope this Essay will tend to elucidate the thought. If they owed their origin entirely to the miasmata of marshes, in such places only should we see them, but they burst forth in populous cities; and here, to suppose the presence of marsh miasmata, would be absurd.

It may be said, perhaps, in opposition to what I have here been advancing, (though I have noticed it elsewhere) that the air does not generate the disease, but merely gives activity to a materies morbi already formed. As this is necessarily a question of general importance, I shall not be able to vindicate myself without touching it. That a state of previous weakness, certainly does predispose to disease, which which may be excited by the stimulus (if I may employ the term) of debilitating causes, no person conversant in the philosophy of medicine can bring evidence to overturn; but, that many are victims, and even martyrs of disease, who were far from being in a valetudinary state, is equally as true; and the particular deducible question is, How far are intermittents, owing to a materies morbi, applied from the atmosphere, or to a certain degree of stimulus, furnished by it for putting into action a pro tempore constitutional morbid diathesis? Allowing the atmosphere to possess materies morbi, it might be contended that it would affect all indiscriminately; but against this it may, with legitimate fairness be urged, that some individuals, from idiosyncrasy or from other causes, resist all the sources of known and prevailing contagion; added to which, if the exciting cause was not in the air, there would be a particular known state of the constitution, especially pre-disposing to the disease; but the robust and the weak, alike experience its attacks. Upon chronic weakness, the application of cold, from its debilitating nature, will tend further to weaken; or will cause *prostration* of *strength* to degenerate into chronic weakness; but all these, upon the analogical scale of reasoning, are of an equal balance. That cold should have the effect of causing a reiterated ague in a body previously weakened, both from its own peculiar nature, and its known effects, is nothing extraordinary. It is from hence plain, that the ague returning from exposure to a bleak wind, is nothing but a secondary effect; but that which is first created by the subtraction of the vivifying principle of the air, is the primary cause of the whole subsequent motions.

Since the derangement of the animal œconomy, with regard to intermittents, is so well understood, and as the prevention of the accession of a paroxysm helps to the cure and eventually confirms it; every thing in the least conducive to so desirable a purpose should be assiduously employed. The adage; "Si quid movendum, principio movendum," forms here an additional and very weighty argument, since this type of disease is very apt to degenerate into inveterate debility, or else to precede one of the modifications of dropsy. The removal of the atony from the extreme vessels being our acknowledged object, would not the inspiration of oxygen gas, as being auxiliary to this effect, be most useful? Might not the atmosphere of the sick person's chamber be purified with oxygen, artificially made, made, or the constant introduction of fresh air from without. And it remains a question, if those things which furnish this aërial principle in abundance, (such as those used in scorbutics) not taken in drachms or ounces, but in quarts, would not tend much to the convalescence of our patient? The more I reflect on these questions, the better I am convinced that they are perfectly apposite to the curative agency; and since in an incipient ague, they may theoretically be supposed equal to its cure; when used conjointly with the medicine mostly employed, they constitute a method of pre-eminent efficiency. It is more particularly in this way, that I think I may venture to recommend this method. Allow me not to be understood to have it in intention to cram the stomach of our patient; the mere inspiration of oxygen with the accustomed medicines, seem to me sufficient.

With those who regard this Essay, more as the visionary speculations of the theorist, than as suggestions of practical good, this mode of practice can have in itself nothing exceptionable. The diffusion of oxygen, by its stimulus, helps to awaken the sensorial power, and overcome the defective excitement; the operation of the usual medicines has the same tendency; thus, whether we attempt to reconcile the means of cure with the present refinement, or in the present adopted doctrine, we must allow the perfect coincidence of both.

I am, &c.

*March*, 16, 1807. J. W. O.

